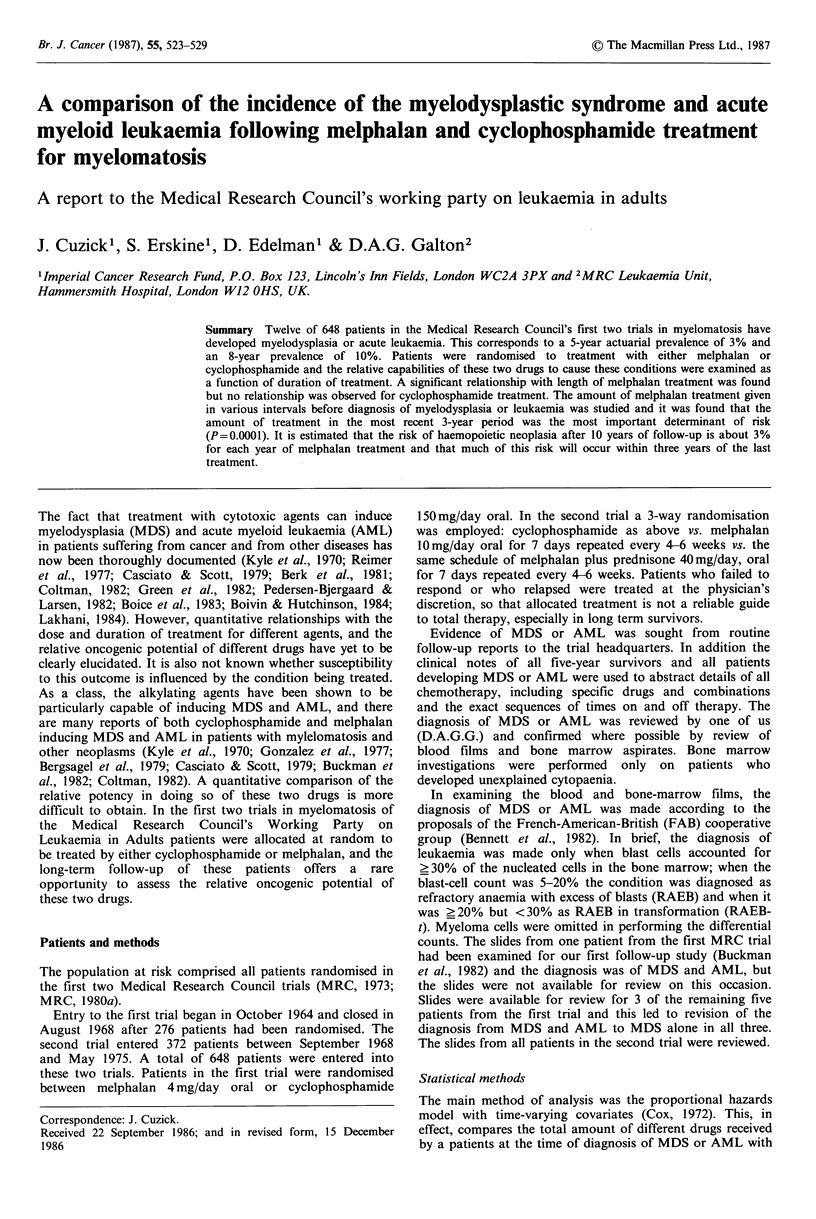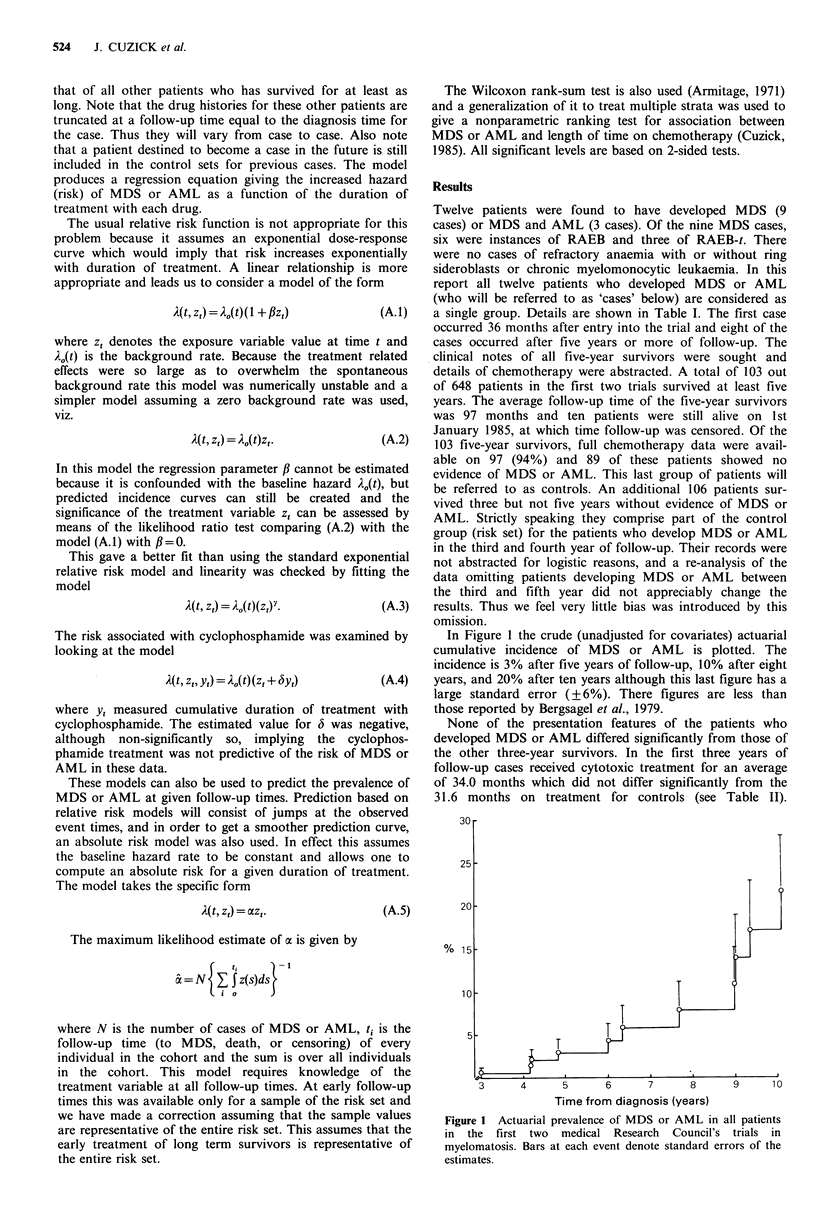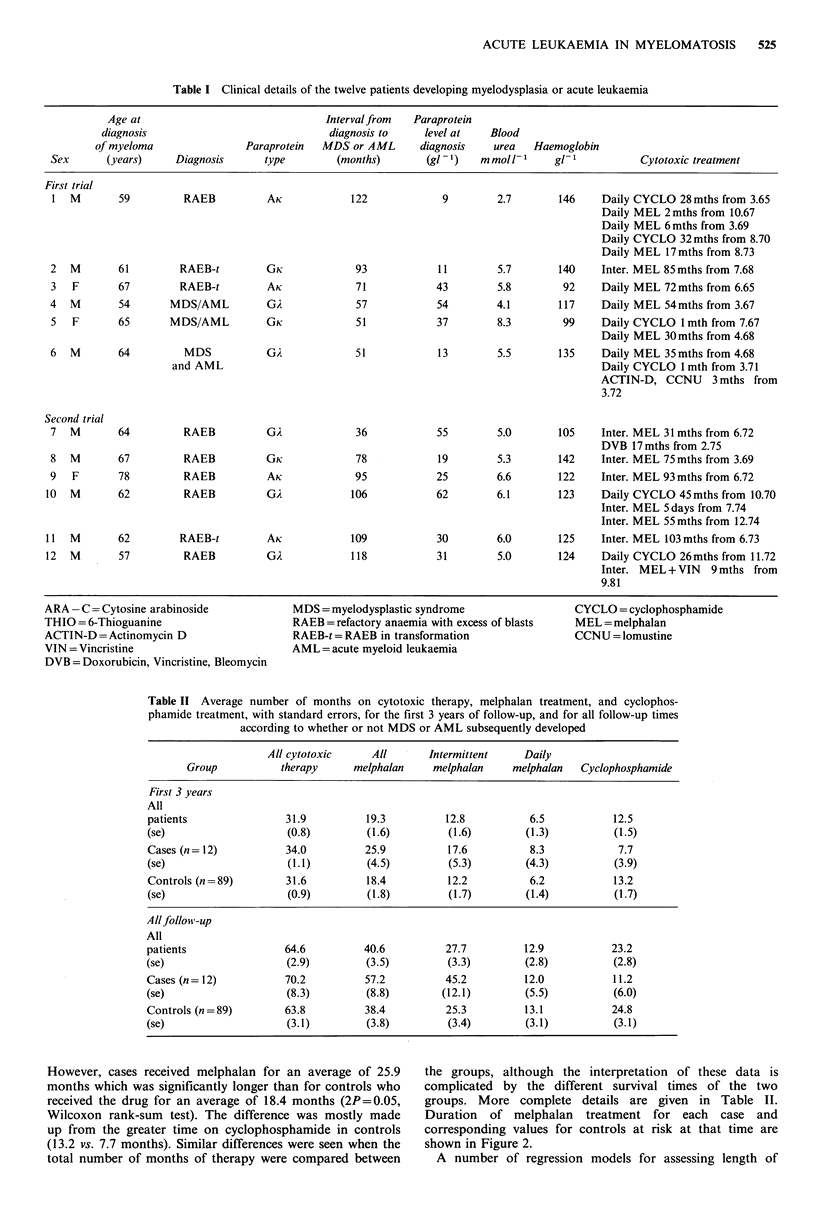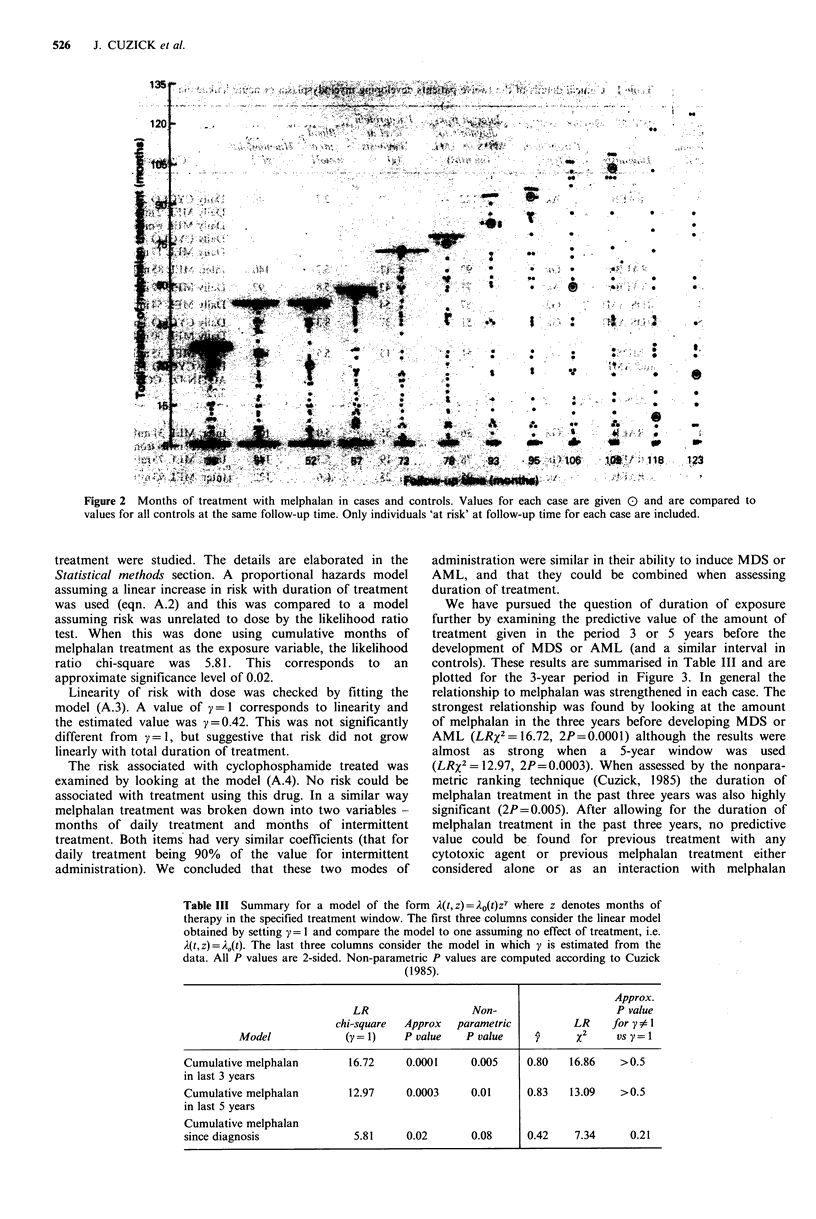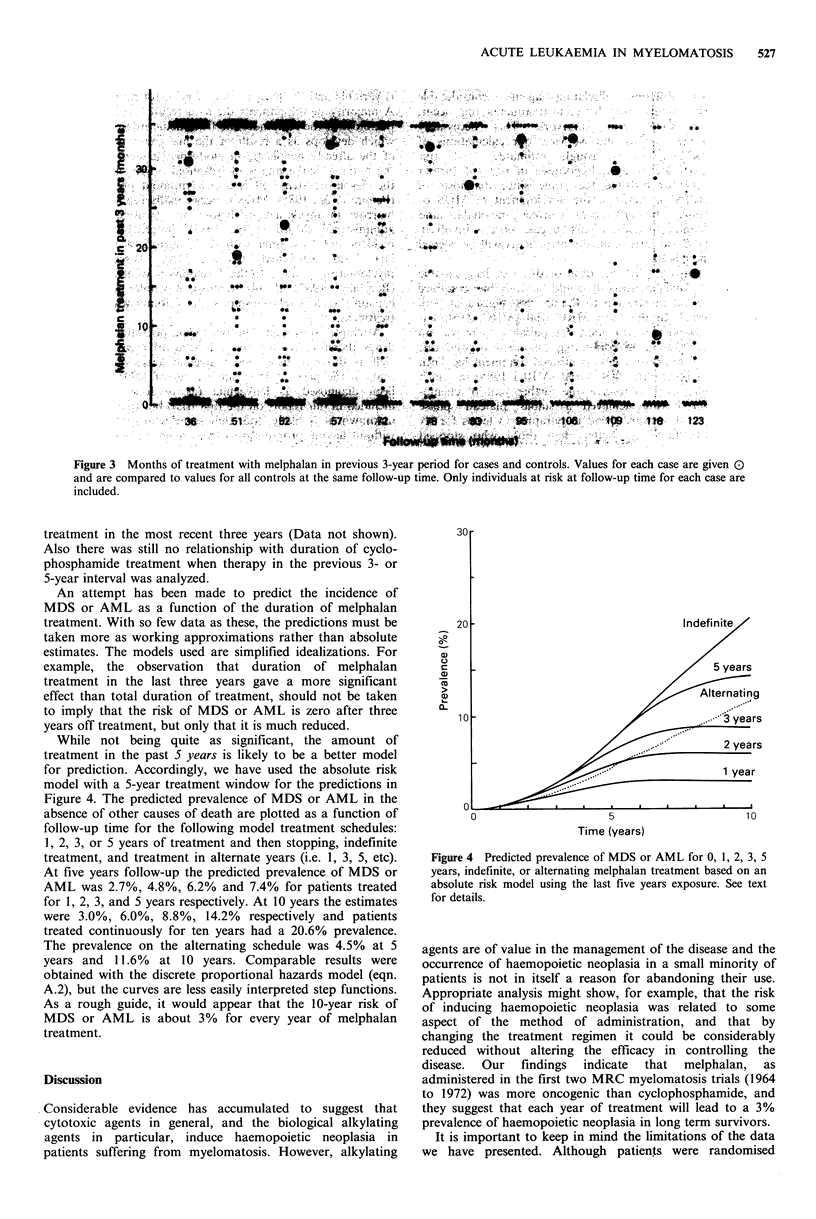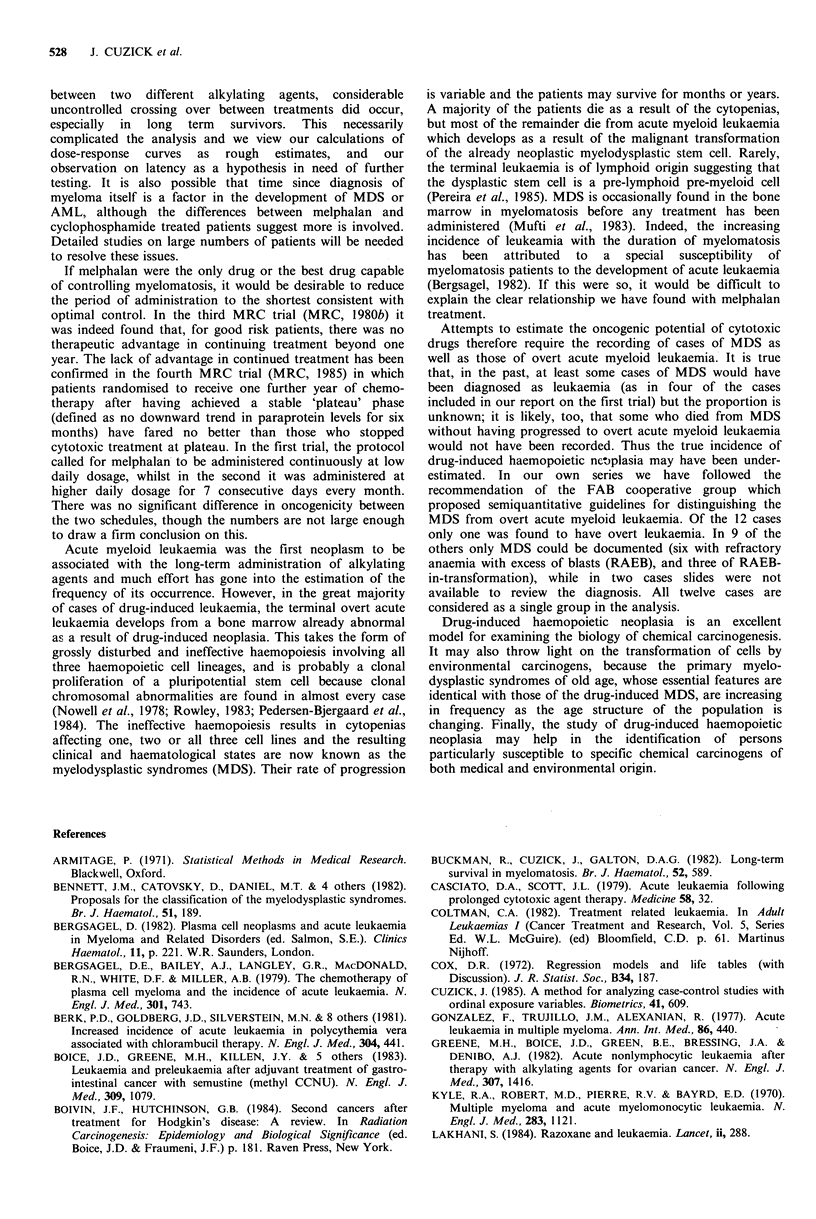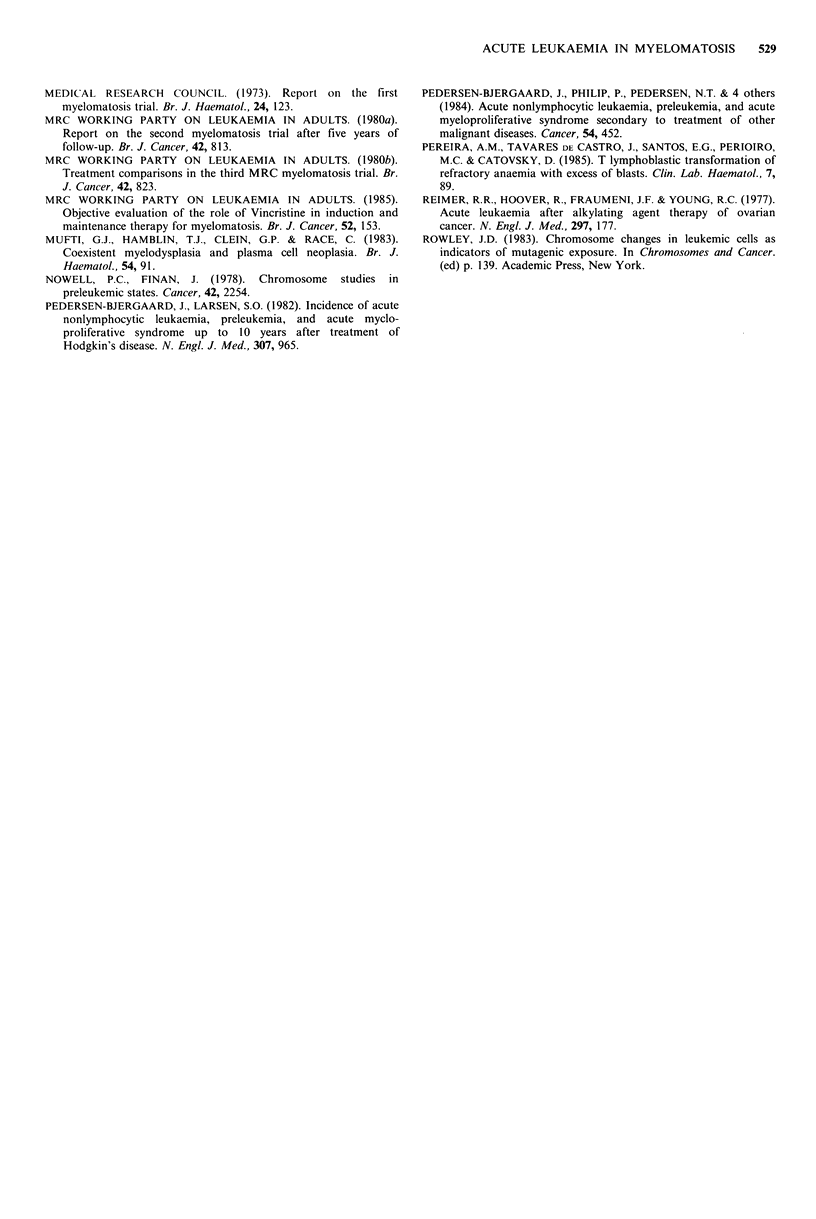# A comparison of the incidence of the myelodysplastic syndrome and acute myeloid leukaemia following melphalan and cyclophosphamide treatment for myelomatosis. A report to the Medical Research Council's working party on leukaemia in adults.

**DOI:** 10.1038/bjc.1987.107

**Published:** 1987-05

**Authors:** J. Cuzick, S. Erskine, D. Edelman, D. A. Galton

## Abstract

Twelve of 648 patients in the Medical Research Council's first two trials in myelomatosis have developed myelodysplasia or acute leukaemia. This corresponds to a 5-year actuarial prevalence of 3% and an 8-year prevalence of 10%. Patients were randomised to treatment with either melphalan or cyclophosphamide and the relative capabilities of these two drugs to cause these conditions were examined as a function of duration of treatment. A significant relationship with length of melphalan treatment was found but no relationship was observed for cyclophosphamide treatment. The amount of melphalan treatment given in various intervals before diagnosis of myelodysplasia or leukaemia was studied and it was found that the amount of treatment in the most recent 3-year period was the most important determinant of risk (P = 0.0001). It is estimated that the risk of haemopoietic neoplasia after 10 years of follow-up is about 3% for each year of melphalan treatment and that much of this risk will occur within three years of the last treatment.


 © The Macmillan Press Ltd., 1987

### A comparison of the incidence of the myelodysplastic syndrome and acute myeloid leukaemia following melphalan and cyclophosphamide treatment for myelomatosis

### A report to the Medical Research Council's working party on leukaemia in adults

J. Cuzick[1], S. Erskine[1], D. Edelman[1] & D.A.G. Galton[2]

[1]*Imperial Cancer Research Fund, P.O. Box 123, Lincoln's Inn Fields, London WC2A 3PX and* [2]*MRC Leukaemia Unit, Hammersmith Hospital, London W12 0HS, UK.*

**Summary** Twelve of 648 patients in the Medical Research Council's first two trials in myelomatosis have developed myelodysplasia or acute leukaemia. This corresponds to a 5-year actuarial prevalence of 3% and an 8-year prevalence of 10%. Patients were randomised to treatment with either melphalan or cyclophosphamide and the relative capabilities of these two drugs to cause these conditions were examined as a function of duration of treatment. A significant relationship with length of melphalan treatment was found but no relationship was observed for cyclophosphamide treatment. The amount of melphalan treatment given in various intervals before diagnosis of myelodysplasia or leukaemia was studied and it was found that the amount of treatment in the most recent 3-year period was the most important determinant of risk ($P=0.0001$). It is estimated that the risk of haemopoietic neoplasia after 10 years of follow-up is about 3% for each year of melphalan treatment and that much of this risk will occur within three years of the last treatment.

The fact that treatment with cytotoxic agents can induce myelodysplasia (MDS) and acute myeloid leukaemia (AML) in patients suffering from cancer and from other diseases has now been thoroughly documented (Kyle *et al*., 1970; Reimer *et al*., 1977; Casciato & Scott, 1979; Berk *et al*., 1981; Coltman, 1982; Green *et al*., 1982; Pedersen-Bjergaard & Larsen, 1982; Boice *et al*., 1983; Boivin & Hutchinson, 1984; Lakhani, 1984). However, quantitative relationships with the dose and duration of treatment for different agents, and the relative oncogenic potential of different drugs have yet to be clearly elucidated. It is also not known whether susceptibility to this outcome is influenced by the condition being treated. As a class, the alkylating agents have been shown to be particularly capable of inducing MDS and AML, and there are many reports of both cyclophosphamide and melphalan inducing MDS and AML in patients with mylelomatosis and other neoplasms (Kyle *et al*., 1970; Gonzalez *et al*., 1977; Bergsagel *et al*., 1979; Casciato & Scott, 1979; Buckman *et al*., 1982; Coltman, 1982). A quantitative comparison of the relative potency in doing so of these two drugs is more difficult to obtain. In the first two trials in myelomatosis of the Medical Research Council's Working Party on Leukaemia in Adults patients were allocated at random to be treated by either cyclophosphamide or melphalan, and the long-term follow-up of these patients offers a rare opportunity to assess the relative oncogenic potential of these two drugs.

### Patients and methods

The population at risk comprised all patients randomised in the first two Medical Research Council trials (MRC, 1973; MRC, 1980*a*).

Entry to the first trial began in October 1964 and closed in August 1968 after 276 patients had been randomised. The second trial entered 372 patients between September 1968 and May 1975. A total of 648 patients were entered into these two trials. Patients in the first trial were randomised between melphalan 4 mg/day oral or cyclophosphamide 150 mg/day oral. In the second trial a 3-way randomisation was employed: cyclophosphamide as above *vs*. melphalan 10 mg/day oral for 7 days repeated every 4–6 weeks *vs*. the same schedule of melphalan plus prednisone 40 mg/day, oral for 7 days repeated every 4–6 weeks. Patients who failed to respond or who relapsed were treated at the physician's discretion, so that allocated treatment is not a reliable guide to total therapy, especially in long term survivors.

Evidence of MDS or AML was sought from routine follow-up reports to the trial headquarters. In addition the clinical notes of all five-year survivors and all patients developing MDS or AML were used to abstract details of all chemotherapy, including specific drugs and combinations and the exact sequences of times on and off therapy. The diagnosis of MDS or AML was reviewed by one of us (D.A.G.G.) and confirmed where possible by review of blood films and bone marrow aspirates. Bone marrow investigations were performed only on patients who developed unexplained cytopaenia.

In examining the blood and bone-marrow films, the diagnosis of MDS or AML was made according to the proposals of the French-American-British (FAB) cooperative group (Bennett *et al*., 1982). In brief, the diagnosis of leukaemia was made only when blast cells accounted for $\geq 30\%$ of the nucleated cells in the bone marrow; when the blast-cell count was 5–20% the condition was diagnosed as refractory anaemia with excess of blasts (RAEB) and when it was $\geq 20\%$ but $<30\%$ as RAEB in transformation (RAEB-*t*). Myeloma cells were omitted in performing the differential counts. The slides from one patient from the first MRC trial had been examined for our first follow-up study (Buckman *et al*., 1982) and the diagnosis was of MDS and AML, but the slides were not available for review on this occasion. Slides were available for review for 3 of the remaining five patients from the first trial and this led to revision of the diagnosis from MDS and AML to MDS alone in all three. The slides from all patients in the second trial were reviewed.

#### *Statistical methods*

The main method of analysis was the proportional hazards model with time-varying covariates (Cox, 1972). This, in effect, compares the total amount of different drugs received by a patients at the time of diagnosis of MDS or AML with

Correspondence: J. Cuzick.
Received 22 September 1986; and in revised form, 15 December 1986

that of all other patients who has survived for at least as long. Note that the drug histories for these other patients are truncated at a follow-up time equal to the diagnosis time for the case. Thus they will vary from case to case. Also note that a patient destined to become a case in the future is still included in the control sets for previous cases. The model produces a regression equation giving the increased hazard (risk) of MDS or AML as a function of the duration of treatment with each drug.

The usual relative risk function is not appropriate for this problem because it assumes an exponential dose-response curve which would imply that risk increases exponentially with duration of treatment. A linear relationship is more appropriate and leads us to consider a model of the form

$$\lambda(t,z_t)=\lambda_o(t)(1+\beta z_t) \quad \text{(A.1)}$$

where $z_t$ denotes the exposure variable value at time $t$ and $\lambda_o(t)$ is the background rate. Because the treatment related effects were so large as to overwhelm the spontaneous background rate this model was numerically unstable and a simpler model assuming a zero background rate was used, viz.

$$\lambda(t,z_t)=\lambda_o(t)z_t. \quad \text{(A.2)}$$

In this model the regression parameter $\beta$ cannot be estimated because it is confounded with the baseline hazard $\lambda_o(t)$, but predicted incidence curves can still be created and the significance of the treatment variable $z_t$ can be assessed by means of the likelihood ratio test comparing (A.2) with the model (A.1) with $\beta=0$.

This gave a better fit than using the standard exponential relative risk model and linearity was checked by fitting the model

$$\lambda(t,z_t)=\lambda_o(t)(z_t)^\gamma. \quad \text{(A.3)}$$

The risk associated with cyclophosphamide was examined by looking at the model

$$\lambda(t,z_t,y_t)=\lambda_o(t)(z_t+\delta y_t) \quad \text{(A.4)}$$

where $y_t$ measured cumulative duration of treatment with cyclophosphamide. The estimated value for $\delta$ was negative, although non-significantly so, implying the cyclophosphamide treatment was not predictive of the risk of MDS or AML in these data.

These models can also be used to predict the prevalence of MDS or AML at given follow-up times. Prediction based on relative risk models will consist of jumps at the observed event times, and in order to get a smoother prediction curve, an absolute risk model was also used. In effect this assumes the baseline hazard rate to be constant and allows one to compute an absolute risk for a given duration of treatment. The model takes the specific form

$$\lambda(t,z_t)=\alpha z_t. \quad \text{(A.5)}$$

The maximum likelihood estimate of $\alpha$ is given by

$$\hat{\alpha}=N\left\{\sum_i \int_o^{t_i} z(s)ds\right\}^{-1}$$

where $N$ is the number of cases of MDS or AML, $t_i$ is the follow-up time (to MDS, death, or censoring) of every individual in the cohort and the sum is over all individuals in the cohort. This model requires knowledge of the treatment variable at all follow-up times. At early follow-up times this was available only for a sample of the risk set and we have made a correction assuming that the sample values are representative of the entire risk set. This assumes that the early treatment of long term survivors is representative of the entire risk set.

The Wilcoxon rank-sum test is also used (Armitage, 1971) and a generalization of it to treat multiple strata was used to give a nonparametric ranking test for association between MDS or AML and length of time on chemotherapy (Cuzick, 1985). All significant levels are based on 2-sided tests.

### Results

Twelve patients were found to have developed MDS (9 cases) or MDS and AML (3 cases). Of the nine MDS cases, six were instances of RAEB and three of RAEB-*t*. There were no cases of refractory anaemia with or without ring sideroblasts or chronic myelomonocytic leukaemia. In this report all twelve patients who developed MDS or AML (who will be referred to as 'cases' below) are considered as a single group. Details are shown in Table I. The first case occurred 36 months after entry into the trial and eight of the cases occurred after five years or more of follow-up. The clinical notes of all five-year survivors were sought and details of chemotherapy were abstracted. A total of 103 out of 648 patients in the first two trials survived at least five years. The average follow-up time of the five-year survivors was 97 months and ten patients were still alive on 1st January 1985, at which time follow-up was censored. Of the 103 five-year survivors, full chemotherapy data were available on 97 (94%) and 89 of these patients showed no evidence of MDS or AML. This last group of patients will be referred to as controls. An additional 106 patients survived three but not five years without evidence of MDS or AML. Strictly speaking they comprise part of the control group (risk set) for the patients who develop MDS or AML in the third and fourth year of follow-up. Their records were not abstracted for logistic reasons, and a re-analysis of the data omitting patients developing MDS or AML between the third and fifth year did not appreciably change the results. Thus we feel very little bias was introduced by this omission.

In Figure 1 the crude (unadjusted for covariates) actuarial cumulative incidence of MDS or AML is plotted. The incidence is 3% after five years of follow-up, 10% after eight years, and 20% after ten years although this last figure has a large standard error (±6%). There figures are less than those reported by Bergsagel *et al.*, 1979.

None of the presentation features of the patients who developed MDS or AML differed significantly from those of the other three-year survivors. In the first three years of follow-up cases received cytotoxic treatment for an average of 34.0 months which did not differ significantly from the 31.6 months on treatment for controls (see Table II).

**Figure 1** Actuarial prevalence of MDS or AML in all patients in the first two medical Research Council's trials in myelomatosis. Bars at each event denote standard errors of the estimates.

**Table I** Clinical details of the twelve patients developing myelodysplasia or acute leukaemia

| | Sex | *Age at diagnosis of myeloma (years)* | *Diagnosis* | *Paraprotein type* | *Interval from diagnosis to MDS or AML (months)* | *Paraprotein level at diagnosis* ($gl^{-1}$) | *Blood urea* $m\,mol\,l^{-1}$ | *Haemoglobin* $gl^{-1}$ | *Cytotoxic treatment* |
|---|---|---|---|---|---|---|---|---|---|
| *First trial* | | | | | | | | | |
| 1 | M | 59 | RAEB | Aκ | 122 | 9 | 2.7 | 146 | Daily CYCLO 28 mths from 3.65<br>Daily MEL 2 mths from 10.67<br>Daily MEL 6 mths from 3.69<br>Daily CYCLO 32 mths from 8.70<br>Daily MEL 17 mths from 8.73 |
| 2 | M | 61 | RAEB-*t* | Gκ | 93 | 11 | 5.7 | 140 | Inter. MEL 85 mths from 7.68 |
| 3 | F | 67 | RAEB-*t* | Aκ | 71 | 43 | 5.8 | 92 | Daily MEL 72 mths from 6.65 |
| 4 | M | 54 | MDS/AML | Gλ | 57 | 54 | 4.1 | 117 | Daily MEL 54 mths from 3.67 |
| 5 | F | 65 | MDS/AML | Gκ | 51 | 37 | 8.3 | 99 | Daily CYCLO 1 mth from 7.67<br>Daily MEL 30 mths from 4.68 |
| 6 | M | 64 | MDS and AML | Gλ | 51 | 13 | 5.5 | 135 | Daily MEL 35 mths from 4.68<br>Daily CYCLO 1 mth from 3.71<br>ACTIN-D, CCNU 3 mths from 3.72 |
| *Second trial* | | | | | | | | | |
| 7 | M | 64 | RAEB | Gλ | 36 | 55 | 5.0 | 105 | Inter. MEL 31 mths from 6.72<br>DVB 17 mths from 2.75 |
| 8 | M | 67 | RAEB | Gκ | 78 | 19 | 5.3 | 142 | Inter. MEL 75 mths from 3.69 |
| 9 | F | 78 | RAEB | Aκ | 95 | 25 | 6.6 | 122 | Inter. MEL 93 mths from 6.72 |
| 10 | M | 62 | RAEB | Gλ | 106 | 62 | 6.1 | 123 | Daily CYCLO 45 mths from 10.70<br>Inter. MEL 5 days from 7.74<br>Inter. MEL 55 mths from 12.74 |
| 11 | M | 62 | RAEB-*t* | Aκ | 109 | 30 | 6.0 | 125 | Inter. MEL 103 mths from 6.73 |
| 12 | M | 57 | RAEB | Gλ | 118 | 31 | 5.0 | 124 | Daily CYCLO 26 mths from 11.72<br>Inter. MEL + VIN 9 mths from 9.81 |

ARA – C = Cytosine arabinoside
THIO = 6-Thioguanine
ACTIN-D = Actinomycin D
VIN = Vincristine
DVB = Doxorubicin, Vincristine, Bleomycin
MDS = myelodysplastic syndrome
RAEB = refactory anaemia with excess of blasts
RAEB-*t* = RAEB in transformation
AML = acute myeloid leukaemia
CYCLO = cyclophosphamide
MEL = melphalan
CCNU = lomustine

**Table II** Average number of months on cytotoxic therapy, melphalan treatment, and cyclophosphamide treatment, with standard errors, for the first 3 years of follow-up, and for all follow-up times according to whether or not MDS or AML subsequently developed

| *Group* | *All cytotoxic therapy* | *All melphalan* | *Intermittent melphalan* | *Daily melphalan* | *Cyclophosphamide* |
|---|---|---|---|---|---|
| *First 3 years* | | | | | |
| All patients | 31.9 | 19.3 | 12.8 | 6.5 | 12.5 |
| (se) | (0.8) | (1.6) | (1.6) | (1.3) | (1.5) |
| Cases (*n* = 12) | 34.0 | 25.9 | 17.6 | 8.3 | 7.7 |
| (se) | (1.1) | (4.5) | (5.3) | (4.3) | (3.9) |
| Controls (*n* = 89) | 31.6 | 18.4 | 12.2 | 6.2 | 13.2 |
| (se) | (0.9) | (1.8) | (1.7) | (1.4) | (1.7) |
| *All follow-up* | | | | | |
| All patients | 64.6 | 40.6 | 27.7 | 12.9 | 23.2 |
| (se) | (2.9) | (3.5) | (3.3) | (2.8) | (2.8) |
| Cases (*n* = 12) | 70.2 | 57.2 | 45.2 | 12.0 | 11.2 |
| (se) | (8.3) | (8.8) | (12.1) | (5.5) | (6.0) |
| Controls (*n* = 89) | 63.8 | 38.4 | 25.3 | 13.1 | 24.8 |
| (se) | (3.1) | (3.8) | (3.4) | (3.1) | (3.1) |

However, cases received melphalan for an average of 25.9 months which was significantly longer than for controls who received the drug for an average of 18.4 months ($2P = 0.05$, Wilcoxon rank-sum test). The difference was mostly made up from the greater time on cyclophosphamide in controls (13.2 *vs.* 7.7 months). Similar differences were seen when the total number of months of therapy were compared between the groups, although the interpretation of these data is complicated by the different survival times of the two groups. More complete details are given in Table II. Duration of melphalan treatment for each case and corresponding values for controls at risk at that time are shown in Figure 2.

A number of regression models for assessing length of

**Figure 2** Months of treatment with melphalan in cases and controls. Values for each case are given ⊙ and are compared to values for all controls at the same follow-up time. Only individuals 'at risk' at follow-up time for each case are included.

treatment were studied. The details are elaborated in the *Statistical methods* section. A proportional hazards model assuming a linear increase in risk with duration of treatment was used (eqn. A.2) and this was compared to a model assuming risk was unrelated to dose by the likelihood ratio test. When this was done using cumulative months of melphalan treatment as the exposure variable, the likelihood ratio chi-square was 5.81. This corresponds to an approximate significance level of 0.02.

Linearity of risk with dose was checked by fitting the model (A.3). A value of $\gamma=1$ corresponds to linearity and the estimated value was $\gamma=0.42$. This was not significantly different from $\gamma=1$, but suggestive that risk did not grow linearly with total duration of treatment.

The risk associated with cyclophosphamide treated was examined by looking at the model (A.4). No risk could be associated with treatment using this drug. In a similar way melphalan treatment was broken down into two variables – months of daily treatment and months of intermittent treatment. Both items had very similar coefficients (that for daily treatment being 90% of the value for intermittent administration). We concluded that these two modes of administration were similar in their ability to induce MDS or AML, and that they could be combined when assessing duration of treatment.

We have pursued the question of duration of exposure further by examining the predictive value of the amount of treatment given in the period 3 or 5 years before the development of MDS or AML (and a similar interval in controls). These results are summarised in Table III and are plotted for the 3-year period in Figure 3. In general the relationship to melphalan was strengthened in each case. The strongest relationship was found by looking at the amount of melphalan in the three years before developing MDS or AML ($LR\chi^2=16.72$, $2P=0.0001$) although the results were almost as strong when a 5-year window was used ($LR\chi^2=12.97$, $2P=0.0003$). When assessed by the nonparametric ranking technique (Cuzick, 1985) the duration of melphalan treatment in the past three years was also highly significant ($2P=0.005$). After allowing for the duration of melphalan treatment in the past three years, no predictive value could be found for previous treatment with any cytotoxic agent or previous melphalan treatment either considered alone or as an interaction with melphalan

**Table III** Summary for a model of the form $\lambda(t,z)=\lambda_0(t)z^\gamma$ where $z$ denotes months of therapy in the specified treatment window. The first three columns consider the linear model obtained by setting $\gamma=1$ and compare the model to one assuming no effect of treatment, i.e. $\lambda(t,z)=\lambda_0(t)$. The last three columns consider the model in which $\gamma$ is estimated from the data. All $P$ values are 2-sided. Non-parametric $P$ values are computed according to Cuzick (1985).

| *Model* | *LR chi-square* ($\gamma=1$) | *Approx P value* | *Non-parametric P value* | $\hat{\gamma}$ | *LR* $\chi^2$ | *Approx. P value for* $\gamma\neq1$ *vs* $\gamma=1$ |
|---|---|---|---|---|---|---|
| Cumulative melphalan in last 3 years | 16.72 | 0.0001 | 0.005 | 0.80 | 16.86 | >0.5 |
| Cumulative melphalan in last 5 years | 12.97 | 0.0003 | 0.01 | 0.83 | 13.09 | >0.5 |
| Cumulative melphalan since diagnosis | 5.81 | 0.02 | 0.08 | 0.42 | 7.34 | 0.21 |

**Figure 3** Months of treatment with melphalan in previous 3-year period for cases and controls. Values for each case are given ⊙ and are compared to values for all controls at the same follow-up time. Only individuals at risk at follow-up time for each case are included.

treatment in the most recent three years (Data not shown). Also there was still no relationship with duration of cyclophosphamide treatment when therapy in the previous 3- or 5-year interval was analyzed.

An attempt has been made to predict the incidence of MDS or AML as a function of the duration of melphalan treatment. With so few data as these, the predictions must be taken more as working approximations rather than absolute estimates. The models used are simplified idealizations. For example, the observation that duration of melphalan treatment in the last three years gave a more significant effect than total duration of treatment, should not be taken to imply that the risk of MDS or AML is zero after three years off treatment, but only that it is much reduced.

While not being quite as significant, the amount of treatment in the past *5 years* is likely to be a better model for prediction. Accordingly, we have used the absolute risk model with a 5-year treatment window for the predictions in Figure 4. The predicted prevalence of MDS or AML in the absence of other causes of death are plotted as a function of follow-up time for the following model treatment schedules: 1, 2, 3, or 5 years of treatment and then stopping, indefinite treatment, and treatment in alternate years (i.e. 1, 3, 5, etc). At five years follow-up the predicted prevalence of MDS or AML was 2.7%, 4.8%, 6.2% and 7.4% for patients treated for 1, 2, 3, and 5 years respectively. At 10 years the estimates were 3.0%, 6.0%, 8.8%, 14.2% respectively and patients treated continuously for ten years had a 20.6% prevalence. The prevalence on the alternating schedule was 4.5% at 5 years and 11.6% at 10 years. Comparable results were obtained with the discrete proportional hazards model (eqn. A.2), but the curves are less easily interpreted step functions. As a rough guide, it would appear that the 10-year risk of MDS or AML is about 3% for every year of melphalan treatment.

**Figure 4** Predicted prevalence of MDS or AML for 0, 1, 2, 3, 5 years, indefinite, or alternating melphalan treatment based on an absolute risk model using the last five years exposure. See text for details.

### Discussion

Considerable evidence has accumulated to suggest that cytotoxic agents in general, and the biological alkylating agents in particular, induce haemopoietic neoplasia in patients suffering from myelomatosis. However, alkylating agents are of value in the management of the disease and the occurrence of haemopoietic neoplasia in a small minority of patients is not in itself a reason for abandoning their use. Appropriate analysis might show, for example, that the risk of inducing haemopoietic neoplasia was related to some aspect of the method of administration, and that by changing the treatment regimen it could be considerably reduced without altering the efficacy in controlling the disease. Our findings indicate that melphalan, as administered in the first two MRC myelomatosis trials (1964 to 1972) was more oncogenic than cyclophosphamide, and they suggest that each year of treatment will lead to a 3% prevalence of haemopoietic neoplasia in long term survivors.

It is important to keep in mind the limitations of the data we have presented. Although patients were randomised between two different alkylating agents, considerable uncontrolled crossing over between treatments did occur, especially in long term survivors. This necessarily complicated the analysis and we view our calculations of dose-response curves as rough estimates, and our observation on latency as a hypothesis in need of further testing. It is also possible that time since diagnosis of myeloma itself is a factor in the development of MDS or AML, although the differences between melphalan and cyclophosphamide treated patients suggest more is involved. Detailed studies on large numbers of patients will be needed to resolve these issues.

If melphalan were the only drug or the best drug capable of controlling myelomatosis, it would be desirable to reduce the period of administration to the shortest consistent with optimal control. In the third MRC trial (MRC, 1980*b*) it was indeed found that, for good risk patients, there was no therapeutic advantage in continuing treatment beyond one year. The lack of advantage in continued treatment has been confirmed in the fourth MRC trial (MRC, 1985) in which patients randomised to receive one further year of chemotherapy after having achieved a stable 'plateau' phase (defined as no downward trend in paraprotein levels for six months) have fared no better than those who stopped cytotoxic treatment at plateau. In the first trial, the protocol called for melphalan to be administered continuously at low daily dosage, whilst in the second it was administered at higher daily dosage for 7 consecutive days every month. There was no significant difference in oncogenicity between the two schedules, though the numbers are not large enough to draw a firm conclusion on this.

Acute myeloid leukaemia was the first neoplasm to be associated with the long-term administration of alkylating agents and much effort has gone into the estimation of the frequency of its occurrence. However, in the great majority of cases of drug-induced leukaemia, the terminal overt acute leukaemia develops from a bone marrow already abnormal as a result of drug-induced neoplasia. This takes the form of grossly disturbed and ineffective haemopoiesis involving all three haemopoietic cell lineages, and is probably a clonal proliferation of a pluripotential stem cell because clonal chromosomal abnormalities are found in almost every case (Nowell *et al.*, 1978; Rowley, 1983; Pedersen-Bjergaard *et al.*, 1984). The ineffective haemopoiesis results in cytopenias affecting one, two or all three cell lines and the resulting clinical and haematological states are now known as the myelodysplastic syndromes (MDS). Their rate of progression is variable and the patients may survive for months or years. A majority of the patients die as a result of the cytopenias, but most of the remainder die from acute myeloid leukaemia which develops as a result of the malignant transformation of the already neoplastic myelodysplastic stem cell. Rarely, the terminal leukaemia is of lymphoid origin suggesting that the dysplastic stem cell is a pre-lymphoid pre-myeloid cell (Pereira *et al.*, 1985). MDS is occasionally found in the bone marrow in myelomatosis before any treatment has been administered (Mufti *et al.*, 1983). Indeed, the increasing incidence of leukeamia with the duration of myelomatosis has been attributed to a special susceptibility of myelomatosis patients to the development of acute leukaemia (Bergsagel, 1982). If this were so, it would be difficult to explain the clear relationship we have found with melphalan treatment.

Attempts to estimate the oncogenic potential of cytotoxic drugs therefore require the recording of cases of MDS as well as those of overt acute myeloid leukaemia. It is true that, in the past, at least some cases of MDS would have been diagnosed as leukaemia (as in four of the cases included in our report on the first trial) but the proportion is unknown; it is likely, too, that some who died from MDS without having progressed to overt acute myeloid leukaemia would not have been recorded. Thus the true incidence of drug-induced haemopoietic neoplasia may have been underestimated. In our own series we have followed the recommendation of the FAB cooperative group which proposed semiquantitative guidelines for distinguishing the MDS from overt acute myeloid leukaemia. Of the 12 cases only one was found to have overt leukaemia. In 9 of the others only MDS could be documented (six with refractory anaemia with excess of blasts (RAEB), and three of RAEB-in-transformation), while in two cases slides were not available to review the diagnosis. All twelve cases are considered as a single group in the analysis.

Drug-induced haemopoietic neoplasia is an excellent model for examining the biology of chemical carcinogenesis. It may also throw light on the transformation of cells by environmental carcinogens, because the primary myelodysplastic syndromes of old age, whose essential features are identical with those of the drug-induced MDS, are increasing in frequency as the age structure of the population is changing. Finally, the study of drug-induced haemopoietic neoplasia may help in the identification of persons particularly susceptible to specific chemical carcinogens of both medical and environmental origin.

### References

ARMITAGE, P. (1971). *Statistical Methods in Medical Research.* Blackwell, Oxford.

BENNETT, J.M., CATOVSKY, D., DANIEL, M.T. & 4 others (1982). Proposals for the classification of the myelodysplastic syndromes. *Br. J. Haematol.*, **51**, 189.

BERGSAGEL, D. (1982). Plasma cell neoplasms and acute leukaemia in Myeloma and Related Disorders (ed. Salmon, S.E.). *Clinics Haematol.*, **11**, p. 221. W.R. Saunders, London.

BERGSAGEL, D.E., BAILEY, A.J., LANGLEY, G.R., MACDONALD, R.N., WHITE, D.F. & MILLER, A.B. (1979). The chemotherapy of plasma cell myeloma and the incidence of acute leukaemia. *N. Engl. J. Med.*, **301**, 743.

BERK, P.D., GOLDBERG, J.D., SILVERSTEIN, M.N. & 8 others (1981). Increased incidence of acute leukaemia in polycythemia vera associated with chlorambucil therapy. *N. Engl. J. Med.*, **304**, 441.

BOICE, J.D., GREENE, M.H., KILLEN, J.Y. & 5 others (1983). Leukaemia and preleukaemia after adjuvant treatment of gastrointestinal cancer with semustine (methyl CCNU). *N. Engl. J. Med.*, **309**, 1079.

BOIVIN, J.F., HUTCHINSON, G.B. (1984). Second cancers after treatment for Hodgkin's disease: A review. In *Radiation Carcinogenesis: Epidemiology and Biological Significance* (ed. Boice, J.D. & Fraumeni, J.F.) p. 181. Raven Press, New York.

BUCKMAN, R., CUZICK, J., GALTON, D.A.G. (1982). Long-term survival in myelomatosis. *Br. J. Haematol.*, **52**, 589.

CASCIATO, D.A., SCOTT, J.L. (1979). Acute leukaemia following prolonged cytotoxic agent therapy. *Medicine* **58**, 32.

COLTMAN, C.A. (1982). Treatment related leukaemia. In *Adult Leukaemias I* (Cancer Treatment and Research, Vol. 5, Series Ed. W.L. McGuire). (ed) Bloomfield, C.D. p. 61. Martinus Nijhoff.

COX, D.R. (1972). Regression models and life tables (with Discussion). *J. R. Statist. Soc.*, **B34**, 187.

CUZICK, J. (1985). A method for analyzing case-control studies with ordinal exposure variables. *Biometrics*, **41**, 609.

GONZALEZ, F., TRUJILLO, J.M., ALEXANIAN, R. (1977). Acute leukaemia in multiple myeloma. *Ann. Int. Med.*, **86**, 440.

GREENE, M.H., BOICE, J.D., GREEN, B.E., BRESSING, J.A. & DENIBO, A.J. (1982). Acute nonlymphocytic leukaemia after therapy with alkylating agents for ovarian cancer. *N. Engl. J. Med.*, **307**, 1416.

KYLE, R.A., ROBERT, M.D., PIERRE, R.V. & BAYRD, E.D. (1970). Multiple myeloma and acute myelomonocytic leukaemia. *N. Engl. J. Med.*, **283**, 1121.

LAKHANI, S. (1984). Razoxane and leukaemia. *Lancet*, **ii**, 288.

MEDICAL RESEARCH COUNCIL. (1973). Report on the first myelomatosis trial. *Br. J. Haematol.*, **24**, 123.

MRC WORKING PARTY ON LEUKAEMIA IN ADULTS. (1980*a*). Report on the second myelomatosis trial after five years of follow-up. *Br. J. Cancer*, **42**, 813.

MRC WORKING PARTY ON LEUKAEMIA IN ADULTS. (1980*b*). Treatment comparisons in the third MRC myelomatosis trial. *Br. J. Cancer*, **42**, 823.

MRC WORKING PARTY ON LEUKAEMIA IN ADULTS. (1985). Objective evaluation of the role of Vincristine in induction and maintenance therapy for myelomatosis. *Br. J. Cancer*, **52**, 153.

MUFTI, G.J., HAMBLIN, T.J., CLEIN, G.P. & RACE, C. (1983). Coexistent myelodysplasia and plasma cell neoplasia. *Br. J. Haematol.*, **54**, 91.

NOWELL, P.C., FINAN, J. (1978). Chromosome studies in preleukemic states. *Cancer*, **42**, 2254.

PEDERSEN-BJERGAARD, J., LARSEN, S.O. (1982). Incidence of acute nonlymphocytic leukaemia, preleukemia, and acute myclo-proliferative syndrome up to 10 years after treatment of Hodgkin's disease. *N. Engl. J. Med.*, **307**, 965.

PEDERSEN-BJERGAARD, J., PHILIP, P., PEDERSEN, N.T. & 4 others (1984). Acute nonlymphocytic leukaemia, preleukemia, and acute myeloproliferative syndrome secondary to treatment of other malignant diseases. *Cancer*, **54**, 452.

PEREIRA, A.M., TAVARES DE CASTRO, J., SANTOS, E.G., PERIOIRO, M.C. & CATOVSKY, D. (1985). T lymphoblastic transformation of refractory anaemia with excess of blasts. *Clin. Lab. Haematol.*, **7**, 89.

REIMER, R.R., HOOVER, R., FRAUMENI, J.F. & YOUNG, R.C. (1977). Acute leukaemia after alkylating agent therapy of ovarian cancer. *N. Engl. J. Med.*, **297**, 177.

ROWLEY, J.D. (1983). Chromosome changes in leukemic cells as indicators of mutagenic exposure. In *Chromosomes and Cancer*. (ed) p. 139. Academic Press, New York.